# *cis*-Dichloridobis(2-phenyl­pyridine-κ*N*)platinum(II)

**DOI:** 10.1107/S160053681004393X

**Published:** 2010-10-31

**Authors:** Nobuto Yoshinari, Naoki Kitani, Takumi Konno

**Affiliations:** a Department of Chemistry, Graduate School of Science, Osaka University, Toyonaka, Osaka 560-0043, Japan

## Abstract

In the title complex, *cis*-[PtCl_2_(C_11_H_9_N)_2_], the Pt^II^ ion is situated in a slightly distorted square-planar environment coordinated by two N atoms from two 2-phenyl­pyridine ligands and two Cl atoms. The two pyridyl planes are inclined with dihedral angles of 59.1 (2) and 61.84 (19)° with respect to the PtCl_2_N_2_ plane. In the crystal, the complex mol­ecules display inter- and intra­molecular π–π stacking inter­actions, with centroid-centroid distances of 3.806 (5)–3.845 (5) Å, which form a one-dimensional column structure along the *a* axis.

## Related literature

For an NMR study on the title compound, see: Pazderski *et al.* (2009 ▶). For the crystal structures of closely related metal complexes, see: Chi & Chou (2010 ▶); Evans *et al.* (2006 ▶); Mdleleni *et al.* (1995 ▶); Okada *et al.* (2001 ▶); Saito *et al.* (2010 ▶).
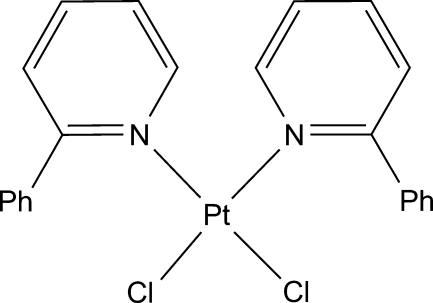


## Experimental

### 

#### Crystal data

[PtCl_2_(C_11_H_9_N)_2_]
*M* *_r_* = 576.37
Monoclinic, 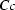

*a* = 7.6457 (8) Å
*b* = 18.0712 (19) Å
*c* = 14.9876 (12) Å
β = 96.014 (7)°
*V* = 2059.4 (3) Å^3^
*Z* = 4
Mo *K*α radiation
μ = 7.08 mm^−1^
*T* = 200 K
0.30 × 0.05 × 0.03 mm

#### Data collection

Rigaku R-AXIS RAPID diffractometer
Absorption correction: multi-scan (*ABSCOR*; Higashi, 1995 ▶) *T*_min_ = 0.329, *T*_max_ = 0.494
9783 measured reflections
4488 independent reflections
3862 reflections with *I* > 2σ(*I*)
*R*_int_ = 0.046

#### Refinement

*R*[*F* ^2^ > 2σ(*F* ^2^)] = 0.039
*wR*(*F* ^2^) = 0.076
*S* = 1.04
4488 reflections
244 parameters
2 restraints
H-atom parameters constrained
Δρ_max_ = 1.71 e Å^−3^
Δρ_min_ = −1.44 e Å^−3^
Absolute structure: Flack (1983 ▶), 2143 Friedel pairs
Flack parameter: 0.010 (10)

### 

Data collection: *PROCESS-AUTO* (Rigaku, 1998 ▶); cell refinement: *PROCESS-AUTO*; data reduction: *CrystalStructure* (Rigaku/MSC, 2004 ▶); program(s) used to solve structure: *SHELXS97* (Sheldrick, 2008 ▶); program(s) used to refine structure: *SHELXL97* (Sheldrick, 2008 ▶); molecular graphics: *Mercury* (Macrae *et al.*, 2006 ▶) and *ORTEP-3* (Farrugia, 1997 ▶); software used to prepare material for publication: *publCIF* (Westrip, 2010 ▶).

## Supplementary Material

Crystal structure: contains datablocks I, global. DOI: 10.1107/S160053681004393X/is2622sup1.cif

Structure factors: contains datablocks I. DOI: 10.1107/S160053681004393X/is2622Isup2.hkl

Additional supplementary materials:  crystallographic information; 3D view; checkCIF report

## Figures and Tables

**Fig. 1.**
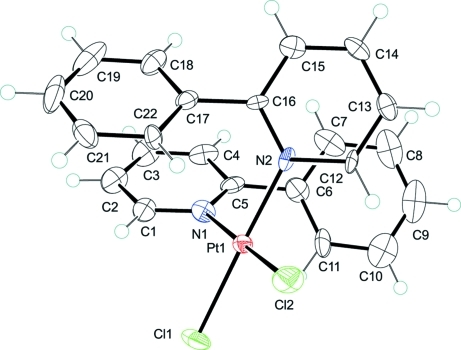
A view of molecular structure of (I), showing the atom-numbering scheme and 50% probability displacement ellipsoids.

**Fig. 2.**
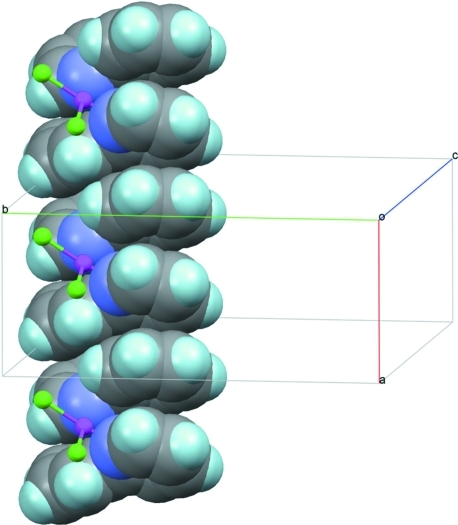
A view of the one-dimensional columnar structure formed along the a axis in (I).

## supplementary crystallographic information

### Comment

It has been well known that metal complexes with 2-phenylpyridinate (ppy = C_11_H_8_N) show intense photoluminescence, especially for Ir^III^ and Pt^II^ complexes (Evans *et al.*, 2006; Chi & Chou, 2010). Recently, we found that the Ir^III^ complex having both ppy and D-Hpen ligands, [Ir^III^(ppy)_2_(D-Hpen)] (D—H_2_pen = *D*-penicillamine), readily reacts with Ag^I^ ion to give a luminescent S bridged Ir^III^Ag^I^Ir^III^ trinuclear complex, [Ag{Ir(ppy)_2_(D—H_0.5_pen)}_2_] (Saito *et al.*, 2010). We report herein the crystal structure of a platinum(II) complex with two monodentate 2-phenylpyridine ligands, [PtCl_2_(C_11_H_9_N)_2_] (I), which was accidentally obtained in the course of the reaction of [PtCl(ppy-*κ*^2^*N*,*C*)]_2_ with 1-thio-β-D-glucose.

The molecular structure of (I) is shown in Fig. 1. In (I), the two pyridyl planes of 2-phenylpyridine ligands are tilted to the coordination plane of Pt1; each of the dihedral angles of the pyridyl unit with respect to the Pt1/N1/N2/Cl1/Cl2 plane is 59.1 (2)° for the N1/C1–C5 plane and 61.84 (19)° for the N2/C12–C16 plane. In each 2-phenylpyridine ligand, the pyridyl and phenyl rings are inclined with angles of 40.4 (2)° for the N1/C1–C5 and C6—C11 planes and 48.1 (2)° for the N2/C12–C16 and C17—C22 planes, allowing them to form a pair of intramolecular π–π stacking interactions with the closest separations of 3.201 (9) and 3.256 (9) Å. Moreover, the complex molecule contacts to the neighboring molecules through intermolecular π–π stacking interactions with the closest separations of 3.438 (10) and 3.389 (10) Å, giving a one-dimensional columnar structure along the *a* axis (Fig. 2).

### Experimental

The reaction of [PtCl(ppy)]_2_ with 1-thio-β-D-glucose sodium salt in ethanol/water (*v*/*v* = 4/1) gave a yellow solution. The reaction solution was evaporated to dryness and was recrystallized from hot ethanol to give a small amount of yellow needle crystals of (I).

### Refinement

H atoms bonded to C atoms were placed at calculated positions and refined as riding, with *U*_iso_(H) = 1.2*U*_eq_(C).

### Crystal data

**Table:**

[PtCl_2_(C_11_H_9_N)_2_]	*F*(000) = 1104
*M**_r_* = 576.37	*D*_x_ = 1.859 Mg m^−^^3^
Monoclinic, *C**c*	Mo *K*α radiation, λ = 0.71075 Å
Hall symbol: C -2yc	Cell parameters from 7887 reflections
*a* = 7.6457 (8) Å	θ = 3.1–27.4°
*b* = 18.0712 (19) Å	µ = 7.08 mm^−^^1^
*c* = 14.9876 (12) Å	*T* = 200 K
β = 96.014 (7)°	Needle, yellow
*V* = 2059.4 (3) Å^3^	0.30 × 0.05 × 0.03 mm
*Z* = 4	

### Data collection

**Table:**

Rigaku R-AXIS RAPID diffractometer	3862 reflections with *I* > 2σ(*I*)
Detector resolution: 10.000 pixels mm^-1^	*R*_int_ = 0.046
ω scans	θ_max_ = 27.4°, θ_min_ = 3.1°
Absorption correction: multi-scan (*ABSCOR*; Higashi, 1995)	*h* = −9→9
*T*_min_ = 0.329, *T*_max_ = 0.494	*k* = −23→23
9783 measured reflections	*l* = −17→19
4488 independent reflections	

### Refinement

**Table:**

Refinement on *F*^2^	Secondary atom site location: difference Fourier map
Least-squares matrix: full	Hydrogen site location: inferred from neighbouring sites
*R*[*F*^2^ > 2σ(*F*^2^)] = 0.039	H-atom parameters constrained
*wR*(*F*^2^) = 0.076	*w* = 1/[σ^2^(*F*_o_^2^) + (0.0331*P*)^2^] where *P* = (*F*_o_^2^ + 2*F*_c_^2^)/3
*S* = 1.04	(Δ/σ)_max_ < 0.001
4488 reflections	Δρ_max_ = 1.71 e Å^−^^3^
244 parameters	Δρ_min_ = −1.44 e Å^−^^3^
2 restraints	Absolute structure: Flack (1983), 2143 Friedel pairs
Primary atom site location: structure-invariant direct methods	Flack parameter: 0.010 (10)

### Special details

**Table:**

Geometry. All e.s.d.'s (except the e.s.d. in the dihedral angle between two l.s. planes) are estimated using the full covariance matrix. The cell e.s.d.'s are taken into account individually in the estimation of e.s.d.'s in distances, angles and torsion angles; correlations between e.s.d.'s in cell parameters are only used when they are defined by crystal symmetry. An approximate (isotropic) treatment of cell e.s.d.'s is used for estimating e.s.d.'s involving l.s. planes.
Refinement. Refinement of *F*^2^ against ALL reflections. The weighted *R*-factor *wR* and goodness of fit *S* are based on *F*^2^, conventional *R*-factors *R* are based on *F*, with *F* set to zero for negative *F*^2^. The threshold expression of *F*^2^ > σ(*F*^2^) is used only for calculating *R*-factors(gt) *etc*. and is not relevant to the choice of reflections for refinement. *R*-factors based on *F*^2^ are statistically about twice as large as those based on *F*, and *R*- factors based on ALL data will be even larger.

### Fractional atomic coordinates and isotropic or equivalent isotropic displacement parameters (Å^2^)

**Table:**

	*x*	*y*	*z*	*U*_iso_*/*U*_eq_	
Pt1	0.74923 (3)	0.137846 (14)	0.68733 (3)	0.01865 (8)	
Cl1	0.6380 (6)	0.14739 (18)	0.8241 (2)	0.0380 (9)	
Cl2	0.9250 (3)	0.03890 (12)	0.73490 (14)	0.0347 (5)	
N1	0.6118 (9)	0.2302 (4)	0.6440 (4)	0.0241 (16)	
C1	0.6562 (11)	0.2917 (5)	0.6883 (6)	0.027 (2)	
H1	0.7275	0.2878	0.7440	0.033*	
C2	0.6038 (13)	0.3623 (5)	0.6577 (7)	0.036 (2)	
H2	0.6377	0.4053	0.6916	0.044*	
C3	0.5000 (13)	0.3672 (6)	0.5756 (8)	0.040 (3)	
H3	0.4642	0.4140	0.5513	0.049*	
C4	0.4511 (12)	0.3037 (5)	0.5310 (6)	0.033 (2)	
H4	0.3808	0.3061	0.4749	0.039*	
C5	0.5031 (11)	0.2352 (5)	0.5670 (5)	0.0252 (19)	
C6	0.4362 (13)	0.1654 (5)	0.5187 (6)	0.032 (2)	
C7	0.4466 (13)	0.1573 (6)	0.4288 (6)	0.040 (3)	
H7	0.4962	0.1954	0.3957	0.047*	
C8	0.3829 (14)	0.0917 (7)	0.3855 (7)	0.049 (3)	
H8	0.3868	0.0860	0.3228	0.059*	
C9	0.3158 (14)	0.0368 (6)	0.4340 (8)	0.053 (3)	
H9	0.2755	−0.0074	0.4042	0.064*	
C10	0.3043 (13)	0.0433 (6)	0.5251 (8)	0.046 (3)	
H10	0.2548	0.0052	0.5582	0.055*	
C11	0.3698 (12)	0.1092 (5)	0.5664 (6)	0.032 (2)	
H11	0.3677	0.1148	0.6293	0.039*	
N2	0.8432 (15)	0.1270 (4)	0.5656 (7)	0.017 (2)	
C12	0.7948 (10)	0.0630 (4)	0.5202 (5)	0.0176 (17)	
H12	0.7352	0.0254	0.5493	0.021*	
C13	0.8316 (11)	0.0526 (5)	0.4328 (5)	0.025 (2)	
H13	0.7997	0.0076	0.4025	0.030*	
C14	0.9150 (11)	0.1079 (5)	0.3899 (5)	0.025 (2)	
H14	0.9378	0.1022	0.3292	0.030*	
C15	0.9638 (11)	0.1707 (5)	0.4360 (5)	0.0244 (19)	
H15	1.0212	0.2091	0.4069	0.029*	
C16	0.9313 (10)	0.1798 (5)	0.5246 (5)	0.0161 (17)	
C17	0.9946 (11)	0.2464 (5)	0.5768 (5)	0.0215 (18)	
C18	0.9717 (11)	0.3163 (5)	0.5396 (6)	0.030 (2)	
H18	0.9115	0.3221	0.4812	0.036*	
C19	1.0357 (14)	0.3774 (6)	0.5868 (9)	0.050 (3)	
H19	1.0216	0.4253	0.5607	0.060*	
C20	1.1213 (15)	0.3692 (6)	0.6731 (9)	0.052 (3)	
H20	1.1631	0.4116	0.7063	0.063*	
C21	1.1455 (12)	0.2999 (6)	0.7105 (6)	0.040 (3)	
H21	1.2048	0.2947	0.7691	0.048*	
C22	1.0845 (11)	0.2382 (5)	0.6636 (5)	0.0249 (19)	
H22	1.1025	0.1903	0.6892	0.030*	

### Atomic displacement parameters (Å^2^)

**Table:**

	*U*^11^	*U*^22^	*U*^33^	*U*^12^	*U*^13^	*U*^23^
Pt1	0.02321 (15)	0.01661 (13)	0.01658 (13)	0.0010 (4)	0.00416 (8)	0.0011 (3)
Cl1	0.054 (2)	0.044 (2)	0.0187 (15)	0.0007 (18)	0.0153 (13)	0.0000 (13)
Cl2	0.0444 (15)	0.0290 (13)	0.0301 (12)	0.0094 (11)	0.0017 (9)	0.0108 (9)
N1	0.018 (4)	0.029 (4)	0.026 (4)	0.006 (3)	0.004 (3)	0.000 (3)
C1	0.021 (5)	0.026 (5)	0.037 (5)	0.003 (4)	0.011 (4)	−0.004 (4)
C2	0.033 (6)	0.026 (5)	0.052 (6)	0.008 (5)	0.014 (4)	0.000 (4)
C3	0.028 (6)	0.025 (6)	0.070 (7)	0.015 (5)	0.016 (5)	0.004 (5)
C4	0.023 (5)	0.039 (6)	0.036 (5)	0.005 (5)	0.006 (4)	0.014 (4)
C5	0.021 (5)	0.026 (5)	0.031 (5)	−0.001 (4)	0.013 (3)	0.006 (4)
C6	0.040 (6)	0.027 (5)	0.027 (5)	0.011 (4)	0.000 (4)	0.003 (4)
C7	0.026 (5)	0.063 (8)	0.029 (5)	0.014 (5)	0.000 (4)	−0.001 (4)
C8	0.041 (7)	0.050 (7)	0.052 (6)	0.022 (6)	−0.019 (5)	−0.015 (6)
C9	0.031 (6)	0.037 (7)	0.085 (9)	0.011 (5)	−0.021 (6)	−0.019 (6)
C10	0.028 (6)	0.041 (7)	0.067 (8)	0.012 (5)	−0.007 (5)	0.004 (5)
C11	0.035 (6)	0.021 (5)	0.042 (6)	0.002 (4)	0.006 (4)	−0.015 (4)
N2	0.021 (5)	0.004 (4)	0.026 (6)	0.006 (3)	−0.001 (4)	0.002 (3)
C12	0.018 (4)	0.007 (4)	0.028 (4)	0.000 (3)	0.002 (3)	−0.005 (3)
C13	0.024 (5)	0.024 (5)	0.028 (5)	0.009 (4)	0.003 (3)	−0.008 (3)
C14	0.027 (5)	0.036 (5)	0.013 (4)	0.008 (4)	0.001 (3)	−0.002 (3)
C15	0.024 (5)	0.025 (5)	0.024 (5)	0.009 (4)	0.002 (3)	0.007 (4)
C16	0.014 (4)	0.018 (5)	0.017 (4)	−0.001 (4)	0.005 (3)	0.006 (3)
C17	0.020 (5)	0.020 (5)	0.026 (4)	−0.002 (4)	0.008 (3)	0.002 (3)
C18	0.024 (5)	0.021 (5)	0.045 (5)	0.001 (4)	0.007 (4)	0.004 (4)
C19	0.029 (6)	0.025 (6)	0.098 (10)	−0.004 (5)	0.018 (6)	0.007 (5)
C20	0.035 (6)	0.038 (7)	0.086 (9)	−0.020 (6)	0.019 (6)	−0.031 (6)
C21	0.032 (6)	0.058 (8)	0.032 (5)	−0.012 (5)	0.013 (4)	−0.022 (5)
C22	0.031 (5)	0.018 (5)	0.026 (5)	−0.001 (4)	0.009 (3)	−0.010 (3)

### Geometric parameters (Å, °)

**Table:**

Pt1—N2	2.039 (10)	C10—H10	0.9500
Pt1—N1	2.041 (7)	C11—H11	0.9500
Pt1—Cl2	2.304 (2)	N2—C16	1.352 (11)
Pt1—Cl1	2.306 (4)	N2—C12	1.373 (11)
N1—C1	1.321 (11)	C12—C13	1.381 (11)
N1—C5	1.353 (10)	C12—H12	0.9500
C1—C2	1.400 (12)	C13—C14	1.381 (12)
C1—H1	0.9500	C13—H13	0.9500
C2—C3	1.396 (15)	C14—C15	1.361 (12)
C2—H2	0.9500	C14—H14	0.9500
C3—C4	1.360 (14)	C15—C16	1.386 (11)
C3—H3	0.9500	C15—H15	0.9500
C4—C5	1.392 (12)	C16—C17	1.489 (11)
C4—H4	0.9500	C17—C18	1.385 (12)
C5—C6	1.515 (12)	C17—C22	1.414 (11)
C6—C7	1.366 (13)	C18—C19	1.374 (14)
C6—C11	1.370 (13)	C18—H18	0.9500
C7—C8	1.413 (15)	C19—C20	1.396 (16)
C7—H7	0.9500	C19—H19	0.9500
C8—C9	1.361 (16)	C20—C21	1.376 (15)
C8—H8	0.9500	C20—H20	0.9500
C9—C10	1.383 (15)	C21—C22	1.374 (12)
C9—H9	0.9500	C21—H21	0.9500
C10—C11	1.409 (13)	C22—H22	0.9500
			
N2—Pt1—N1	90.7 (3)	C6—C11—C10	122.1 (9)
N2—Pt1—Cl2	87.3 (3)	C6—C11—H11	118.9
N1—Pt1—Cl2	175.3 (2)	C10—C11—H11	118.9
N2—Pt1—Cl1	178.4 (3)	C16—N2—C12	119.4 (9)
N1—Pt1—Cl1	89.8 (2)	C16—N2—Pt1	125.3 (7)
Cl2—Pt1—Cl1	92.32 (11)	C12—N2—Pt1	115.0 (7)
C1—N1—C5	118.4 (8)	N2—C12—C13	120.9 (8)
C1—N1—Pt1	115.5 (6)	N2—C12—H12	119.5
C5—N1—Pt1	125.2 (6)	C13—C12—H12	119.5
N1—C1—C2	123.5 (9)	C12—C13—C14	119.5 (8)
N1—C1—H1	118.3	C12—C13—H13	120.2
C2—C1—H1	118.3	C14—C13—H13	120.2
C3—C2—C1	117.6 (10)	C15—C14—C13	118.9 (8)
C3—C2—H2	121.2	C15—C14—H14	120.5
C1—C2—H2	121.2	C13—C14—H14	120.5
C4—C3—C2	118.8 (9)	C14—C15—C16	121.2 (8)
C4—C3—H3	120.6	C14—C15—H15	119.4
C2—C3—H3	120.6	C16—C15—H15	119.4
C3—C4—C5	120.5 (9)	N2—C16—C15	120.0 (9)
C3—C4—H4	119.8	N2—C16—C17	118.8 (8)
C5—C4—H4	119.8	C15—C16—C17	121.2 (8)
N1—C5—C4	121.0 (8)	C18—C17—C22	119.7 (8)
N1—C5—C6	119.9 (8)	C18—C17—C16	120.4 (7)
C4—C5—C6	119.1 (8)	C22—C17—C16	119.8 (7)
C7—C6—C11	119.9 (9)	C19—C18—C17	120.2 (9)
C7—C6—C5	120.6 (9)	C19—C18—H18	119.9
C11—C6—C5	119.6 (8)	C17—C18—H18	119.9
C6—C7—C8	119.3 (10)	C18—C19—C20	119.9 (10)
C6—C7—H7	120.3	C18—C19—H19	120.0
C8—C7—H7	120.3	C20—C19—H19	120.0
C9—C8—C7	119.9 (10)	C21—C20—C19	120.3 (10)
C9—C8—H8	120.1	C21—C20—H20	119.8
C7—C8—H8	120.1	C19—C20—H20	119.8
C8—C9—C10	122.1 (10)	C22—C21—C20	120.4 (9)
C8—C9—H9	118.9	C22—C21—H21	119.8
C10—C9—H9	118.9	C20—C21—H21	119.8
C9—C10—C11	116.7 (11)	C21—C22—C17	119.5 (9)
C9—C10—H10	121.7	C21—C22—H22	120.3
C11—C10—H10	121.7	C17—C22—H22	120.3
			
N2—Pt1—N1—C1	−118.5 (6)	N1—Pt1—N2—C16	55.4 (9)
Cl1—Pt1—N1—C1	63.0 (6)	Cl2—Pt1—N2—C16	−120.4 (9)
N2—Pt1—N1—C5	50.4 (7)	N1—Pt1—N2—C12	−117.8 (7)
Cl1—Pt1—N1—C5	−128.1 (7)	Cl2—Pt1—N2—C12	66.4 (7)
C5—N1—C1—C2	−3.3 (12)	C16—N2—C12—C13	−1.4 (14)
Pt1—N1—C1—C2	166.4 (7)	Pt1—N2—C12—C13	172.2 (6)
N1—C1—C2—C3	−0.3 (13)	N2—C12—C13—C14	−1.3 (13)
C1—C2—C3—C4	1.8 (14)	C12—C13—C14—C15	2.0 (12)
C2—C3—C4—C5	0.3 (14)	C13—C14—C15—C16	0.0 (12)
C1—N1—C5—C4	5.5 (12)	C12—N2—C16—C15	3.4 (14)
Pt1—N1—C5—C4	−163.1 (6)	Pt1—N2—C16—C15	−169.5 (7)
C1—N1—C5—C6	−174.5 (8)	C12—N2—C16—C17	−175.5 (8)
Pt1—N1—C5—C6	16.9 (11)	Pt1—N2—C16—C17	11.5 (13)
C3—C4—C5—N1	−4.0 (13)	C14—C15—C16—N2	−2.8 (13)
C3—C4—C5—C6	175.9 (8)	C14—C15—C16—C17	176.2 (8)
N1—C5—C6—C7	−130.7 (9)	N2—C16—C17—C18	−135.0 (9)
C4—C5—C6—C7	49.3 (12)	C15—C16—C17—C18	46.0 (11)
N1—C5—C6—C11	47.3 (12)	N2—C16—C17—C22	47.4 (12)
C4—C5—C6—C11	−132.7 (9)	C15—C16—C17—C22	−131.6 (8)
C11—C6—C7—C8	2.1 (14)	C22—C17—C18—C19	−0.1 (13)
C5—C6—C7—C8	−179.9 (8)	C16—C17—C18—C19	−177.7 (8)
C6—C7—C8—C9	−1.5 (14)	C17—C18—C19—C20	−1.1 (14)
C7—C8—C9—C10	1.2 (16)	C18—C19—C20—C21	1.5 (16)
C8—C9—C10—C11	−1.5 (15)	C19—C20—C21—C22	−0.5 (15)
C7—C6—C11—C10	−2.5 (15)	C20—C21—C22—C17	−0.7 (13)
C5—C6—C11—C10	179.5 (8)	C18—C17—C22—C21	1.1 (12)
C9—C10—C11—C6	2.1 (14)	C16—C17—C22—C21	178.7 (8)

## References

[bb1] Chi, Y. & Chou, P.-T. (2010). *Chem. Soc. Rev.* **39**, 638–655. 10.1039/b916237b 20111785

[bb2] Evans, R. C., Douglas, P. & Winscom, C. J. (2006). *Coord. Chem. Rev.* **250**, 2093–2126.

[bb3] Farrugia, L. J. (1997). *J. Appl. Cryst.* **30**, 565.

[bb4] Flack, H. D. (1983). *Acta Cryst.* A**39**, 876–881.

[bb5] Higashi, T. (1995). *ABSCOR* Rigaku Corporation, Tokyo, Japan.

[bb6] Macrae, C. F., Edgington, P. R., McCabe, P., Pidcock, E., Shields, G. P., Taylor, R., Towler, M. & van de Streek, J. (2006). *J. Appl. Cryst.* **39**, 453–457.

[bb7] Mdleleni, M. M., Bridgewater, J. S., Watts, R. J. & Ford, P. C. (1995). *Inorg* *Chem* **34**, 2334–2342.

[bb8] Okada, T., El-Mehasseb, I. M., Kodaka, M., Tomohiro, T., Okamoto, K. & Okuno, H. (2001). *J. Med. Chem* **44**, 4661–4667. 10.1021/jm010203d 11741483

[bb9] Pazderski, L., Toušek, J., Sitkowski, J., Kozerski, L. & Szłyk, E. (2009). *Magn. Reson. Chem.* **47**, 658–665. 10.1002/mrc.2445 19472306

[bb10] Rigaku (1998). *PROCESS-AUTO* Rigaku Corporation, Tokyo, Japan.

[bb11] Rigaku/MSC (2004). *CrystalStructure* Rigaku/MSC, The Woodlands, Texas, USA.

[bb12] Saito, K., Sarukawa, Y., Tsuge, K. & Konno, T. (2010). *Eur. J. Inorg. Chem.* **25**, 3909–3913.

[bb13] Sheldrick, G. M. (2008). *Acta Cryst.* A**64**, 112–122. 10.1107/S0108767307043930 18156677

[bb14] Westrip, S. P. (2010). *J. Appl. Cryst.* **43**, 920–925.

